# Comprehensive identification of maize ZmE2F transcription factors and the positive role of *ZmE2F6* in response to drought stress

**DOI:** 10.1186/s12864-024-10369-0

**Published:** 2024-05-13

**Authors:** Yang Cao, Kexin Wang, Fengzhong Lu, Qi Li, Qingqing Yang, Bingliang Liu, Hayderbinkhalid Muhammad, Yingge Wang, Fengling Fu, Wanchen Li, Haoqiang Yu

**Affiliations:** 1 Maize Research Institute, Sichuan Agricultural University, Chengdu, 611130 China; 2 College of Food and Biological Engineering, Chengdu University, Chengdu, 610106 China; 3 National Research Centre of Intercropping, The Islamia University of Bahawalpur, Bahawalpur, 63100 Pakistan

**Keywords:** Maize, E2F, Transcription factor, Expression, Drought stress

## Abstract

**Background:**

The early 2 factor (E2F) family is characterized as a kind of transcription factor that plays an important role in cell division, DNA damage repair, and cell size regulation. However, its stress response has not been well revealed.

**Results:**

In this study, ZmE2F members were comprehensively identified in the maize genome, and 21 *ZmE2F* genes were identified, including eight *E2F* subclade members, seven *DEL* subfamily genes, and six DP genes. All ZmE2F proteins possessed the DNA-binding domain (DBD) characterized by conserved motif 1 with the RRIYD sequence. The ZmE2F genes were unevenly distributed on eight maize chromosomes, showed diversity in gene structure, expanded by gene duplication, and contained abundant stress-responsive elements in their promoter regions. Subsequently, the *ZmE2F6* gene was cloned and functionally verified in drought response. The results showed that the ZmE2F6 protein interacted with ZmPP2C26, localized in the nucleus, and responded to drought treatment. The overexpression of *ZmE2F6* enhanced drought tolerance in transgenic *Arabidopsis* with longer root length, higher survival rate, and biomass by upregulating stress-related gene transcription.

**Conclusions:**

This study provides novel insights into a greater understanding and functional study of the E2F family in the stress response.

**Supplementary Information:**

The online version contains supplementary material available at 10.1186/s12864-024-10369-0.

## Background

Environmental stimuli, including drought, salinity, and high temperature, have frequently occurred in recent decades and led to yield loss of crops in agricultural production. Drought stress is a main misfortune in these abiotic stressors and will be a more severe challenge and threat to agriculture and humanity by 2050 [1]. In adaption to drought, plants activate a series of physiological, morphological, and molecular changes [1–3]. Among them, many transcription factors (TFs) can be dominated by stress to regulate gene expression and coordinate plant antagonism to adverse factors [4–6].

The early E2 factor (E2F) family proteins are initially discovered as TFs of the *E2* gene and play key roles in cell proliferation control in adenoviruses [7]. E2Fs can be classified into typical and atypical E2Fs according to protein structure. Typically, E2Fs have only one DNA-binding domain (DBD) and form heterodimeric complexes with dimerization proteins (DPs) to bind the promoters of downstream genes, but atypical E2Fs possess duplicated two DBD [8]. In higher plants, E2Fs are also categorized into three subclades, including E2F, DP, and DEL (DP-E2F-like), due to the difference in the composition of conserved domains [9, 10]. Over the past decade, E2Fs have well been revealed to play significant roles in the cell cycle and DNA damage repair [11–16]. For instance, ectopic expression of *DcE2F1* of *Daucus carota* promotes cell proliferation in *Arabidopsis* seedlings [14]. In *Arabidopsis*, eight E2F members exhibit antagonistic roles in cell proliferation, such as E2Fa/b, which acts as a positive regulator, but E2Fc is a negative regulator [17–19]. E2Fa/b can also activate the DNA damage response and cell cycle progression by differentially regulating the expression of genes [11]. Additionally, AtE2Fa/DPa also inhibits growth, and *AtE2Fa/DPa*-overexpressing plants show an abnormal phenotype owing to ectopic cell division or enhanced DNA endoreduplication [12, 20].

In addition to the crucial role in the cell cycle, few reports show that atypical E2F inhibits the accumulation of salicylic acid to balance growth and defense [21, 22]. Furthermore, AtE2Fa acts downstream of ERECTA kinase, which is involved in cell size and stomatal density [23]. Via expression analysis, it is suggested that *TaE2F-DP* of wheat, *PheE2F/DPs* of Moso bamboo, *E2F/DP* genes of *Medicago truncatula*, and *PvE2F/DPs* of *Phaseolus vulgaris* respond to drought or salt stress, suggesting their potential roles in regulating stress tolerance [9, 24–26]. To date, however, the role of E2F in plant stress tolerance remains obscure.

Maize is a crucial crop worldwide and is widely used as food and livestock feed. During its growth, maize plants show sensitivity to water deficit due to its high water demand, leading to maize yield being greatly affected by drought stress [27–30]. Therefore, it becomes imperative to identify and explore drought tolerance-related genes that can be used to enhance maize resilience through molecular breeding [31–35]. In our previous study, we found that ZmPP2C26 regulated drought tolerance [36] and targeted maize ZmE2F (Zm00001d048412, data not shown), indicating that ZmE2F might be involved in drought response. Hence, in this study, we comprehensively investigated *ZmE2F* genes in the maize genome. Thereafter, phylogenetic relationships, conserved motifs and domains, gene structures and duplication, and protein-protein interaction networks were analyzed. Additionally, the ZmE2F6 (Zm00001d048412) was functionally verified by performing subcellular localization, expression patterns in drought treatment, and ectopic expression in *Arabidopsis* under drought stress. This study will significantly contribute to a better understanding of E2Fs in stress response.

## Methods

### Identification of *ZmE2Fs* in the maize genome

The genome and amino acid data of maize B73 were downloaded from the MaizeGDB database (https://download.maizegdb.org/Zm-B73-REFERENCE-GRAMENE-4.0/). Meanwhile, the coding sequences and amino acid sequences of 8 AtE2Fs and 9 OsE2Fs of *Arabidopsis* and rice were downloaded from the *Arabidopsis* Information Resource (TAIR) (https://www.arabidopsis.org/) and the Rice Genome Annotation Project (RGAP) database (http://rice.uga.edu/) and were used as queries to perform local BLASTp with an E-value of 1e^− 10^ in the maize protein database for maize E2F searching, respectively. After removing the redundant sequences manually, the candidate sequences were further analyzed for the presence of the E2F_DP domain (PF02319) by using PFAM (http://pfam.xfam.org/). The candidates possessing the E2F domain were identified as maize ZmE2F members. The secondary structure and physicochemical properties, including molecular weights, isoelectric point (PI), stability coefficient, and grand average of hydropathicity (GRAVY), of ZmE2Fs were analyzed using SOPMA (https://npsa.lyon.inserm.fr/cgi-bin/npsa_automat.pl?page=/NPSA/npsa_sopma.html) and EXPASY (https://www.expasy.org/). The subcellular localization of ZmE2Fs was predicted using cNLS Mapper (https://nls-mapper.iab.keio.ac.jp/cgi-bin/NLS_Mapper_form.cgi*).*

### Conserved motifs, domains, and phylogenetic analysis

To further identify the conserved motifs and domains, the amino acid sequences of ZmE2Fs were analyzed using MEME (http://meme-suite.org/tools/meme) and NCBI-CDD (https://www.ncbi.nlm.nih.gov/cdd), respectively. The motif and domain composition of each ZmE2F was visualized by TBtools [37]. The protein sequences of all ZmE2F, AtE2F, and OsE2F were multiple-aligned using ClustalW with default parameters. The maximum likelihood tree was built with 1000 bootstrap replications by MEGA11 (https://www.megasoftware.net/). Meanwhile, protein-protein interaction (PPI) analysis among ZmE2F members was performed using the STRING tool [38].

### Gene structure, promoter, duplication, and synteny analyses

The chromosomal location of each *ZmE2F* gene was obtained from the maizeGDB database. The coding sequences and genomic DNA sequences of every *ZmE2F* were downloaded and used to analyze exon-intron composition using Gene Structure Display Server 2.0 (GSDS) (http://gsds.gao-lab.org/). The 2000 bp upstream sequence of the transcription start site of each *ZmE2F* gene was retrieved from maizeGDB and used for *cis*-acting element analysis using PlantCARE (https://bioinformatics.psb.ugent.be/webtools/plantcare/html/). Meanwhile, the gene duplication events and the synteny relationship between *ZmE2F*, *AtE2F*, and *OsE2F* gene members were analyzed using MCScanX with default parameters. The chromosomal location, duplications, and synteny relationships of the *ZmE2F*, *AtE2F*, and *OsE2F* genes were visualized using TBtools [37]. The non-synonymous (Ka) and synonymous (Ks) substitution rates per site of the duplicated gene pairs were calculated using TBtools [37]. Then, the divergence time in millions of years (Mya) was also calculated using the following formula: T = Ks/2λ × 10^− 6^ Mya (λ = 6.5 × 10^− 9^ for grasses) [39].

### Cloning and subcellular localization of the *ZmE2F6* gene

The specific primers (Table S1) were designed by Primer 5.0, synthesized at Tsingke Biotech (Beijing, China), and used to amplify the sequence of the *ZmE2F6* gene from maize B73 cDNA using Phanta Max Super-Fidelity DNA Polymerase (Vazyme, Nanjing). After amplification, the PCR product was purified by a gel recovery kit subcloned, and inserted into the pMD19-T vector to generate pMD19-T-*ZmE2F6* and verified by sequencing. The sequencing result was aligned with the candidate sequence of the *ZmE2F6* gene using DNAMAN. The open reading frame (ORF) sequence of *ZmE2F6* without stop codon was amplified from the pMD19-T-*ZmE2F6* plasmid using the specific primers designed by CE Design V1.04 (Table S1) with the *Xba* I and *Spe* I recognition sites. The PCR products and pCAMBIA2300-*35 S-eGFP* plasmids were digested using *Xba* I and *Spe* I. Subsequently, they were inserted into the *Xba* I and *Spe* I sites of pCAMBIA2300-*35 S-eGFP* to produce the fusion expression vector *35 S-ZmE2F6-eGFP* using the ClonExpress II One Step Cloning Kit (Vazyme, Nanjing). Each construct was introduced into *Agrobacterium tumefaciens* strain GV3101 and then used for transient expression in the leaves of *Nicotiana benthamiana*. As described by Sun et al. [40], the constructs were infiltrated into the leaves of five-week-old *N. benthamiana*. The GFP fluorescence was observed and imaged using a confocal laser scanning microscope (Zeiss 800). The empty vector *35 S-eGFP* was served as the positive control.

### Plant treatment, RNA extraction, and quantitative real-time PCR (qRT‒PCR) analysis

The seeds of maize B73 lines were soaked in 10% H_2_O_2_ for 15 min, rinsed twice using sterile water, soaked in distilled water for 8 h, then wrapped in filter paper and cultured at 28 °C until germination. Subsequently, the seedlings of the same size were transferred to the hydroponic cassette containing hoagland nutrient solution and cultured at 16 h light at 28 °C /8 h dark at 24 °C. Three-leaf-stage seedlings were subjected to 16% PEG-6000 treatment mimicking drought stress, and then shoots containing leaf, stem and leaf sheath, and roots were sampled at 0, 3, 6, 12, and 24 h of treatment, respectively. The total RNA of each sample was extracted using an RNAison plus kit (Takara, Dalian), examined for quality using NanoDrop OneC (ThermoFisher Scientific), treated with DNase to remove DNA contamination, reverse-transcribed into cDNA using a PrimeScript™ RT Regent Kit (Takara, Dalian), and used to perform qRT-PCR. The specific primers of *ZmE2F6* were designed using Primer-BLAST (https://www.ncbi.nlm.nih.gov/tools/primer-blast/index.cgi?LINK_LOC=BlastHome), synthesized at TsingkeBiotech (Beijing, China), and listed in Table S1. The *ZmGAPDH* gene was amplified using specific primers (Table S1) and used as an internal reference. The qRT-PCR was performed in the Bio-Rad CFX96™ Real-Time PCR system using 2 × Universal SYBR Green Fast qPCR Mix (ABclonal, Wuhan). The 20 µL reaction mixture contained 10 µL of 2 × Universal SYBR Green Fast qPCR Mix, 0.4 µL of each forward and reverse primer, 1.0 µL of each cDNA as template, and 8.2 µL of ddH_2_O. The reaction protocol was set as a two-step temperature cycle including 95 °C for 3 min, followed by 40 cycles at 95 °C for 5 s and 60 °C for 30 s. The relative expression level of *ZmE2Fs* was calculated and normalized using the 2^−ΔΔCt^ method [41].

### Y2H and GST pull-down analysis

The ORF of *ZmE2F6* was amplified using specific primers designed by CE Design V1.04 with the *Nde I* and *EcoR I* recognition sites (Table S1) and inserted into the pGADT7 plasmid to generate AD*-ZmE2F6* as described above. The BD-*ZmPP2C26* plasmid was constructed in our previous study [36]. The AD-*ZmE2F6* and BD-*ZmPP2C26* plasmids were cotransformed into the yeast strain Y2H Gold using a yeast transformation kit (Coolaber, Beijing). Subsequently, yeast cells were cultured on synthetic dropout (SD) medium without Trp and Leu (SD/-Trp/-Leu) at 30℃ for 2 days, and then positive clones were transferred onto SD/-Trp/-Leu/-His/-Ade plates with X-α-gal and cultured at 30℃ for 2 days. Meanwhile, the ORF of *ZmE2F6* was amplified and inserted into the *EcoR I* and *Xho I* sites of pGEX-6P-1 to generate *GST*-*ZmE2F6* and used for the GST pull-down assay. The *His*-*ZmPP2C26* plasmids were produced in our previous study. The GST pull-down was performed as described by Lu et al. [36].

### Plant transformation, phenotyping, and RNA-seq

The ORF of *ZmE2F6* was amplified without a stop codon and inserted into the *Xba* I and *Nde* I sites of pRI201-*35 S*-*GUS* to produce *35 S*-*ZmE2F6*-*GUS* as described above. The *35 S*-*ZmE2F6*-*GUS* construct was transformed into *Agrobacterium tumefaciens* strain GV3101 and then used to transform *Arabidopsis thaliana* (Col-0) by the floral-dip method [42]. According to the method of Sun et al. [40], the positive transformants were screened on 1/2 MS plates with 50 mg/L kanamycin, used for harvesting seeds individually. The homozygous lines without segregation on 1/2 MS plates with 50 mg/L kanamycin were screened and used for PCR detection. Meanwhile, the leaves of homozygous lines were sampled and used to perform GUS staining using the GUS Staining Kit (Coolaber, Beijing).

According to the methods described by Sun et al. [40] with minor modifications, for drought stress, the seeds of homozygous lines and wild type (WT) were surface-sterilized, planted on 1/2 MS plates supplemented with 0 (control), 150, and 250 mM mannitol, vernalized for 2 days in the dark at 4 °C, and vertically cultured in a chamber under 10 h light/14 h dark at 22 °C with 60–70% humidity. At 14 days of treatment, the seedlings were photographed and measured for root length. Moreover, another batch of overexpressed lines and WT were sown in soil and incubated in a greenhouse under the same conditions. After 2 weeks, the seedlings were subjected to withholding water for two weeks, then rewatered for at least 2 days, and monitored for phenotyping. Subsequently, the survival number of each line was counted and used to calculate the survival rate. The leaf of each line was sampled, dried at 80 °C for 3 days, and used for biomass measurement.

Meanwhile, three-week-old seedlings of homozygous lines and WT were sampled and used for RNA sequencing at Sanshubio Company (Jiangsu, China). As described by Sun et al. [40], the total RNA of each sample was extracted, qualified for quality and integrity, and used to construct a sequencing library. Then, library sequencing was conducted using the NovaSeq 6000 system. The sequencing adapters and low-quality reads of raw data were removed to generate clean data, which were mapped to the *Arabidopsis* genome by hisat2 [43] and used for assembling transcripts of every gene using StringTie [44]. The differentially expressed genes (DEGs) were confirmed with a p-value < 0.05 and |FoldChange| > 2 using DESeq2 [45]. The GO analysis of DEGs was performed using KOBAS [46].

### Data analysis

All assays were performed with three replicates. The data are shown as the mean values ± standard error (SE). The significance was analyzed by Student’s t-test at the *p* < 0.05 or *p* < 0.01 level.

## Results

### *ZmE2F* members in maize

To identify the maize *ZmE2F* family, a local BLASTp search against the maize protein database was performed using the amino acid sequences of 8 *AtE2F* and 9 *OsE2F* members as query references [10, 47]. As shown in Table 1, a total of 21 ZmE2F members were identified and designated as *ZmE2F1* to *ZmE2F21*. The CDS length of the ZmE2F genes ranged from 648 (*ZmE2F18*) to 1608 bp (*ZmE2F14*), encoding 215 to 535 amino acids with molecular weight varying from 23.39 to 59.21 kDa. The theoretical isoelectric point (pI) of ZmE2F proteins ranged from 4.71 (ZmE2F11) to 9.41 (ZmE2F13), and the grand average hydropathicity (GRAVY) of all ZmE2F proteins was less than 0 and ranged from − 0.062 (ZmE2F1) to -0.603 (ZmE2F5). The instability indices of ZmE2F proteins varied from 31.45 (ZmE2F20) to 62.39 (ZmE2F5), and 19 ZmE2F members contained the highest random coil in their secondary structure, implying that they were unstable hydrophilic proteins. All ZmE2F proteins were predicted to be localized in the nucleus.

**Table 1:** The physical and chemical properties of ZmE2F family

Gene name	Gene ID	CDS length (bp)	Protein length (aa)	Mass (kDa)	PI	II	GRAVY	Secondary structure (%)			Localization
								Alpha helix	Extended strand	Random coil	
*ZmE2F1*	Zm00001d032741	1002	333	37.09	5.99	39.96	-0.062	31.23	18.62	43.84	N
*ZmE2F2*	Zm00001d007384	966	321	34.96	9.01	47.97	-0.441	38.32	13.71	41.12	N
*ZmE2F3*	Zm00001d033566	852	283	33.07	6.60	50.03	-0.533	30.04	21.55	43.11	N
*ZmE2F4*	Zm00001d016737	1392	463	50.56	5.05	53.43	-0.566	26.35	10.80	60.69	N
*ZmE2F5*	Zm00001d004512	1407	468	50.68	5.70	61.97	-0.603	24.36	10.26	62.39	N
*ZmE2F6*	Zm00001d048412	1233	410	44.39	6.02	51.02	-0.570	30.98	11.22	53.90	N
*ZmE2F7*	Zm00001d017986	1344	447	49.25	9.18	41.18	-0.561	37.81	6.94	51.90	N
*ZmE2F8*	Zm00001d016907	852	283	33.23	6.84	51.53	-0.584	33.22	21.20	39.58	N
*ZmE2F9*	Zm00001d011597	759	252	28.38	9.27	48.17	-0.487	47.62	9.13	38.10	N
*ZmE2F10*	Zm00001d045365	732	243	27.19	7.64	40.53	-0.227	33.74	20.99	38.27	N
*ZmE2F11*	Zm00001d023465	1368	455	49.67	4.71	50.12	-0.592	27.91	11.43	57.14	N
*ZmE2F12*	Zm00001d037274	1308	435	48.10	8.80	50.34	-0.567	37.01	7.82	53.33	N
*ZmE2F13*	Zm00001d052288	1335	444	48.70	9.41	45.74	-0.523	40.09	8.11	48.87	N
*ZmE2F14*	Zm00001d038664	1608	535	59.21	8.44	46.46	-0.346	37.57	18.50	35.70	N
*ZmE2F15*	Zm00001d026355	777	258	28.76	6.06	62.33	-0.555	18.60	15.50	59.69	N
*ZmE2F16*	Zm00001d050664	1131	376	40.89	5.35	48.64	-0.401	26.06	13.56	58.78	N
*ZmE2F17*	Zm00001d003755	1104	367	40.73	9.03	43.78	-0.485	29.97	11.44	56.13	N
*ZmE2F18*	Zm00001d014132	648	215	23.39	6.84	57.36	-0.540	43.72	4.19	48.84	N
*ZmE2F19*	Zm00001d027709	1161	386	41.49	6.09	50.25	-0.748	30.05	10.62	55.44	N
*ZmE2F20*	Zm00001d045639	837	278	30.97	8.75	31.45	-0.601	32.73	18.71	44.96	N
*ZmE2F21*	Zm00001d046856	1602	533	58.57	8.55	44.61	-0.224	32.27	21.95	36.77	N

### Phylogenetic analysis

**Fig. 1:**
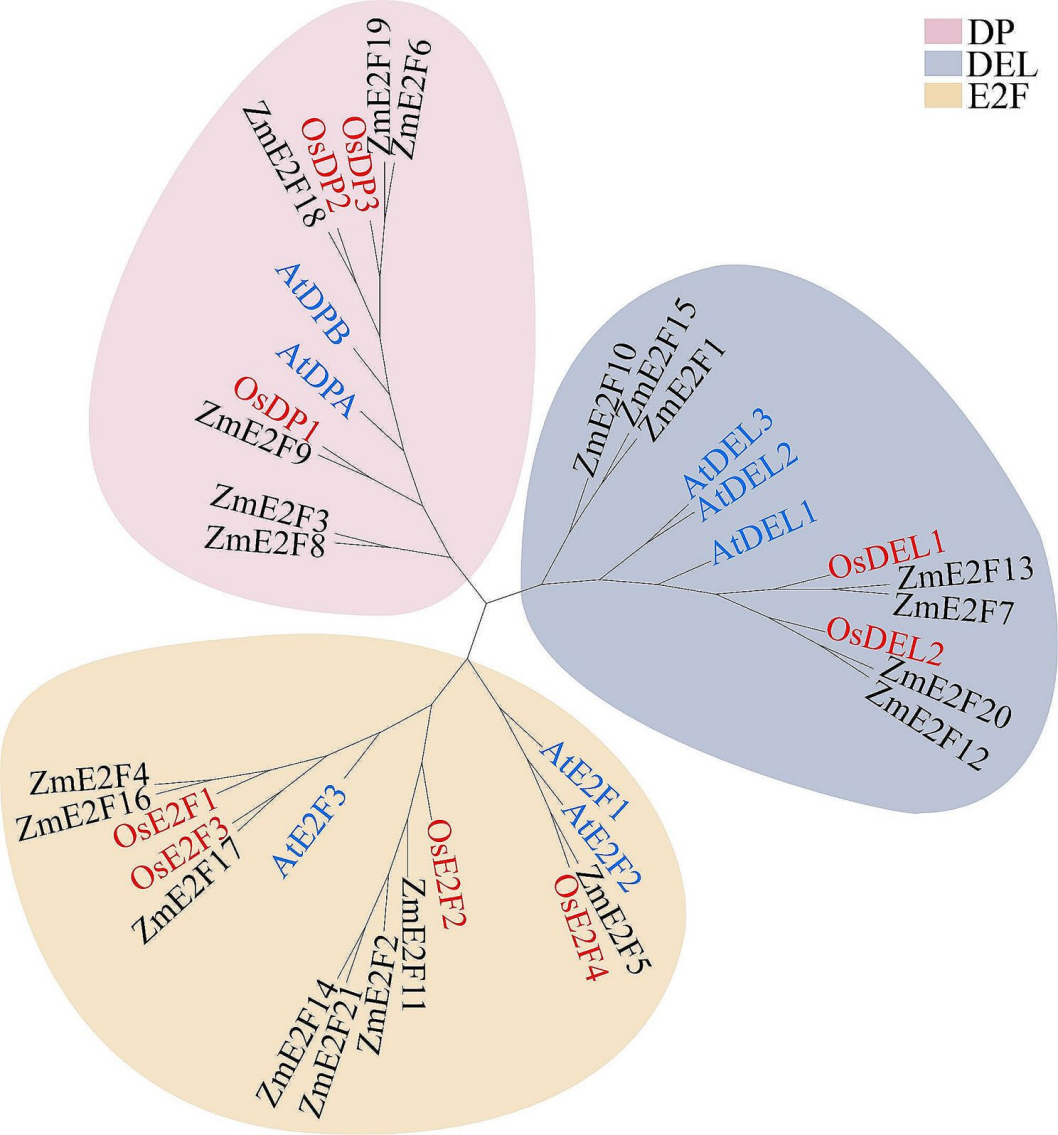
Phylogenetic tree of the E2F family. The E2Fs of *Arabidopsis*, rice, and maize are marked in blue, red, and black, respectively

To investigate the evolutionary relationships of ZmE2Fs and model plants, a total of 38 amino acid sequences, including 21, 8, and 9 E2Fs from maize, *Arabidopsis*, and rice, respectively, were used to construct a phylogenetic tree (Fig. 1). It showed that the ZmE2F proteins could be classified into three subfamilies, including E2F, DP, and DEL clades [9]. Eight members, including ZmE2F2, 4, 5, 11, 14, 16, 17, and 21, were clustered into the E2F subscale with AtE2Fs and OsE2Fs. Seven proteins containing ZmE2F1, 7, 10, 12, 13, 15, and 20 were branched into the DEL subfamily with AtDELs and OsDELs. The other 6 ZmE2Fs (ZmE2F3, 6, 8, 9, 18, and 19) belonged to the DP subclade with AtDP and OsDP members. In addition, ZmE2Fs showed a closer phylogenetic relationship with OsE2Fs than with AtE2Fs, indicating more sequence similarity with OsE2Fs.

### Conserved motif and domain

**Fig. 2:**
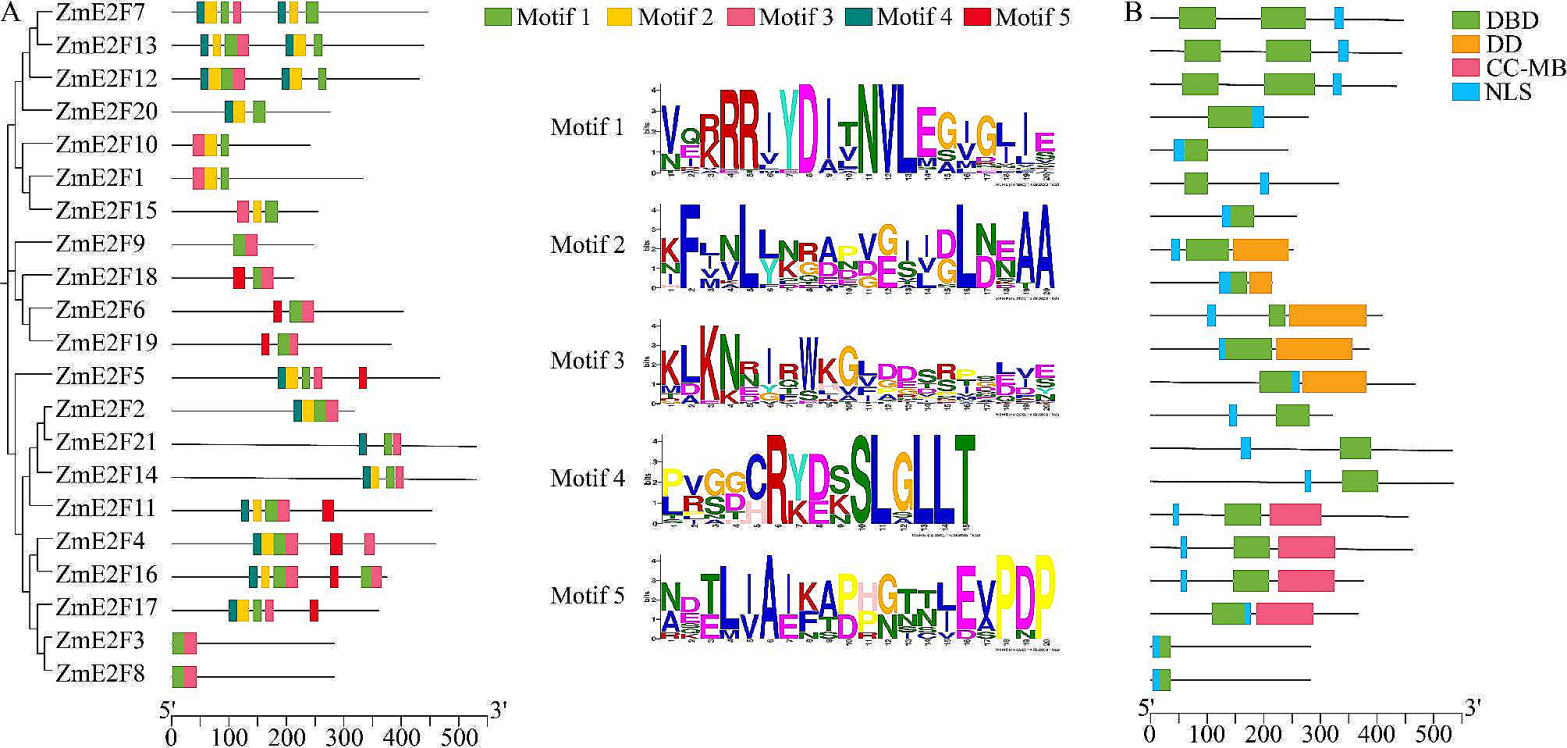
Conserved motif and domain of ZmE2Fs

Five conserved motifs were identified in the amino acid sequences of ZmE2Fs using the MEME online program (Fig. 2A). Among them, motif 1 was highly conserved in all ZmE2F members and characterized by the RRIYD sequence that was a DNA-binding motif followed by dimerization residues DNVLE sequence [48]. Conserved domain analysis revealed that all ZmE2F members contained at least one DNA-binding domain (DBD) (Pfam ID PF02319, Table S2). ZmE2F7, 12, and 13 possessed two DBDs (Fig. 2B). In addition, ZmE2F5, 6, 9, 18, and 19 also contained a dimerization domain (DD). The coiled coil (CC)-marked box (MB) domain (CC-MB) was found in ZmE2F4, 11, 16, and 17. Furthermore, all ZmE2F members contained a nuclear localization signal.

### ZmE2Fs protein-protein Interaction Prediction

**Fig. 3:**
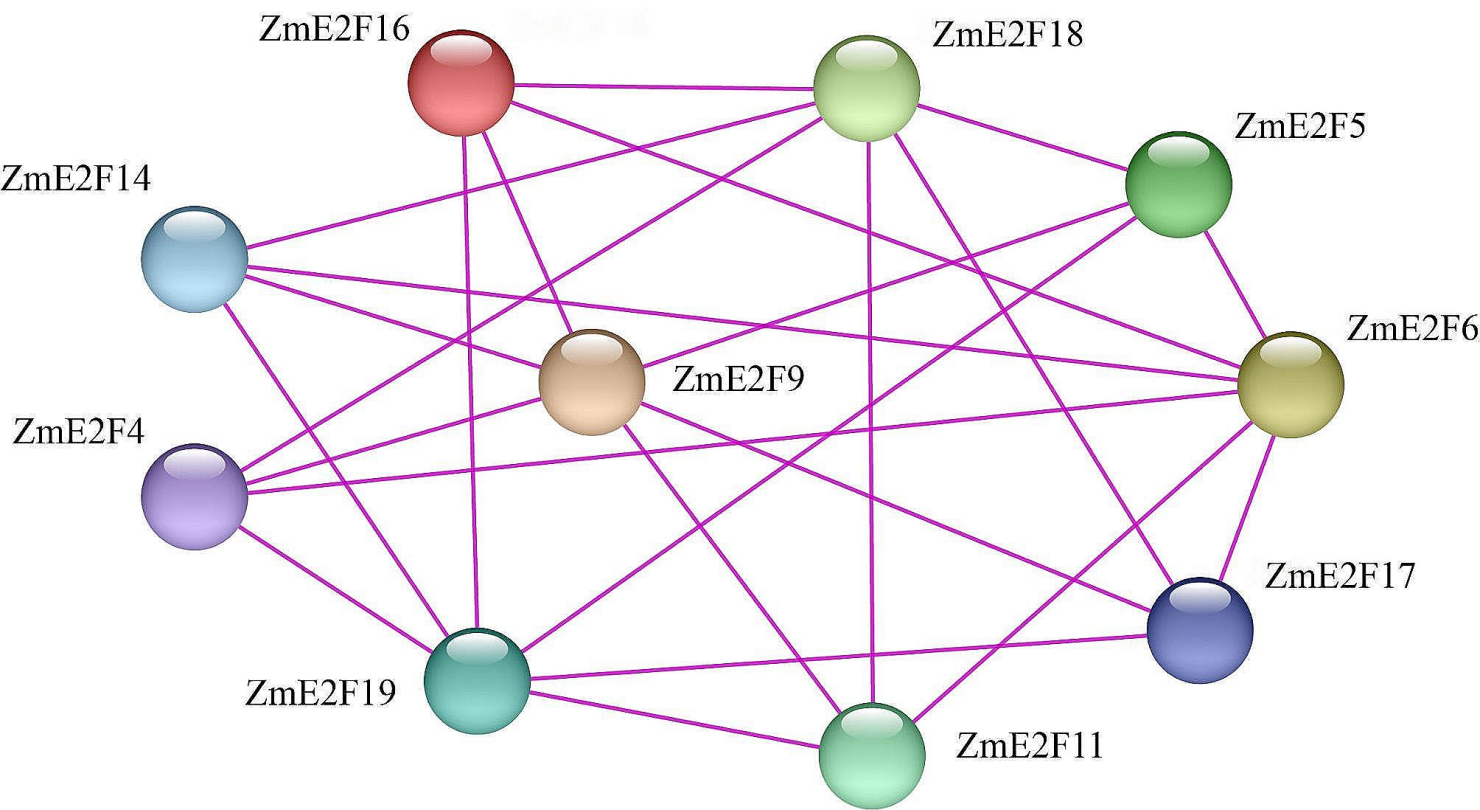
ZmE2F protein-protein interaction prediction

Due to the presence of dimerization residues DNVLE sequence within motif 1 in every ZmE2F and DD or CC-MB in some ZmE2Fs, to explore the potential interactions among ZmE2F members, protein-protein interaction (PPI) analysis was performed. As shown in Fig. 3, ten ZmE2Fs were predicted to interact with each other, which generated 23 PPI combinations. Among them, ZmE2F6, 9, 18 and 19 had the largest number of PPIs (6 interactions), while ZmE2F4, 5, 11, 14, 16, and 17 had 4 PPIs. These results imply that the DNVLE sequence, DD, and CC-MB play crucial roles during ZmE2F dimerization.

### Gene structure, duplication, synteny, and *cis* -acting elements

**Fig. 4:**
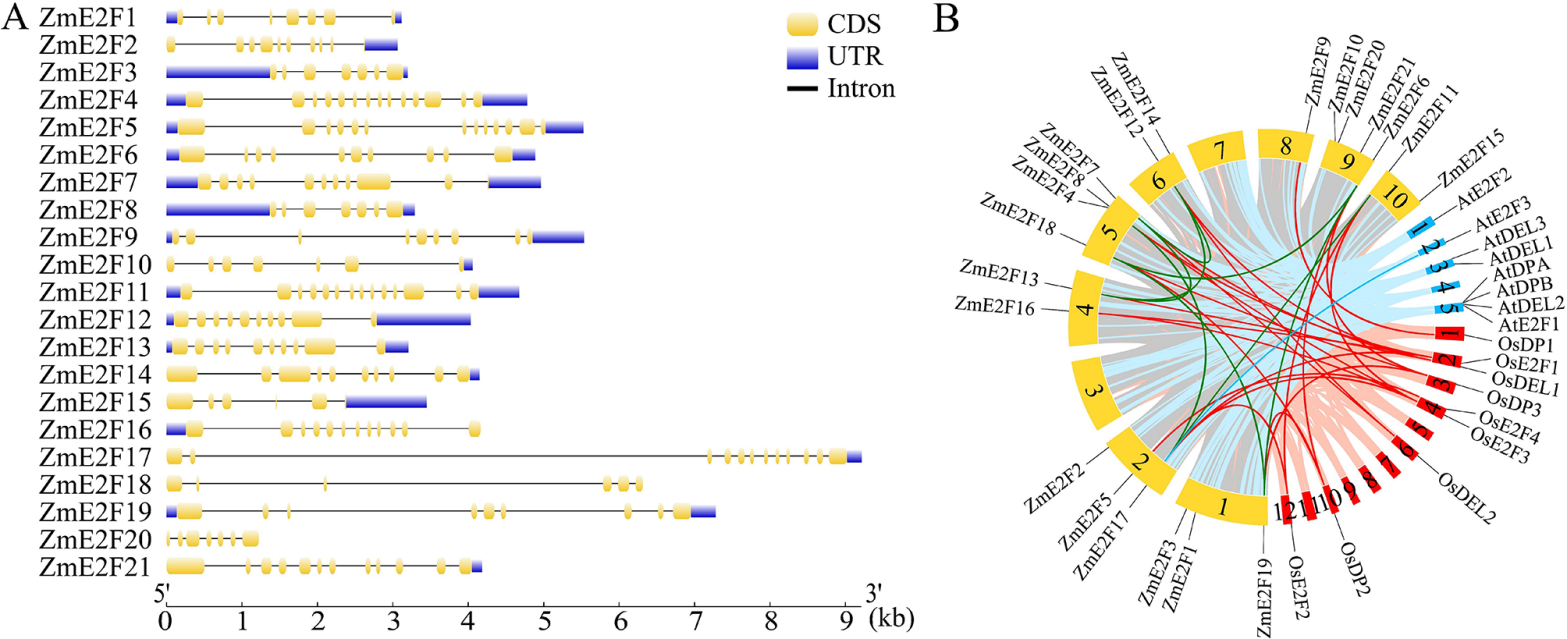
Gene structure (**A**) and duplication (**B**) of ZmE2Fs. Yellow and blue boxes represent exons and untranslated regions, respectively. Black lines represent introns. Yellow, blue, and red boxes with a number represent chromosomes of maize, *Arabidopsis*, and rice, respectively. The green, blue, and red lines indicate duplicated *E2F* gene pairs among maize, as well as between maize and *Arabidopsis* and rice, respectively

Gene structure analysis revealed that the *ZmE2F* gene family showed diversity in exon and intron composition (Fig. 4A). The number of exons among *ZmE2Fs* ranged from six to fourteen. Except for *ZmE2F18* and *ZmE2F20*, the other 19 *ZmE2Fs* contained 5ʹ or 3ʹ terminal untranslated regions. The *ZmE2Fs* were unevenly distributed on eight maize chromosomes, excluding chromosomes 3 and 7. Gene duplication analysis showed that there were eight paralogous pairs of *ZmE2Fs* in the maize genome, including *ZmE2F4* and *ZmE2F17*, *ZmE2F6* and *ZmE2F18*, *ZmE2F6* and *ZmE2F19*, *ZmE2F7* and *ZmE2F12*, *ZmE2F7* and *ZmE2F13*, *ZmE2F11* and *ZmE2F17*, *ZmE2F12* and *ZmE2F13*, and *ZmE2F18* and *ZmE2F19*. Likewise, gene synteny analysis revealed one and twenty-two orthologous pairs between *ZmE2F* and *AtE2F* and *ZmE2F* and *OsE2F*, respectively (Fig. 4B; Table S3). Meanwhile, the Ka/Ks rates among *ZmE2Fs* paralogous pairs ranged from 0.19 to 0.35 and their duplication time was estimated to be 16.54–139.30 million years ago (Table S4). The results suggest that gene duplication contributed to *E2F* expansion during the evolutionary process.

Furthermore, *cis*-acting element analysis revealed that abundant stress- or hormone-responsive elements were found in the promoter regions of *ZmE2Fs*, such as ABREs, MBSs, AREs, and LTR elements (Table S5), which were responsive to abscisic acid, drought, anaerobic induction, and low temperature. For instance, ABREs were found in all *ZmE2F* promoters. MBS elements, the MYB binding site involved in drought response, were found in 15 *ZmE2F* promoters. This finding implies that the *ZmE2F* genes may be involved in abiotic stress responses.

### ZmE2F6 interacts with ZmPP2C26

**Fig. 5:**
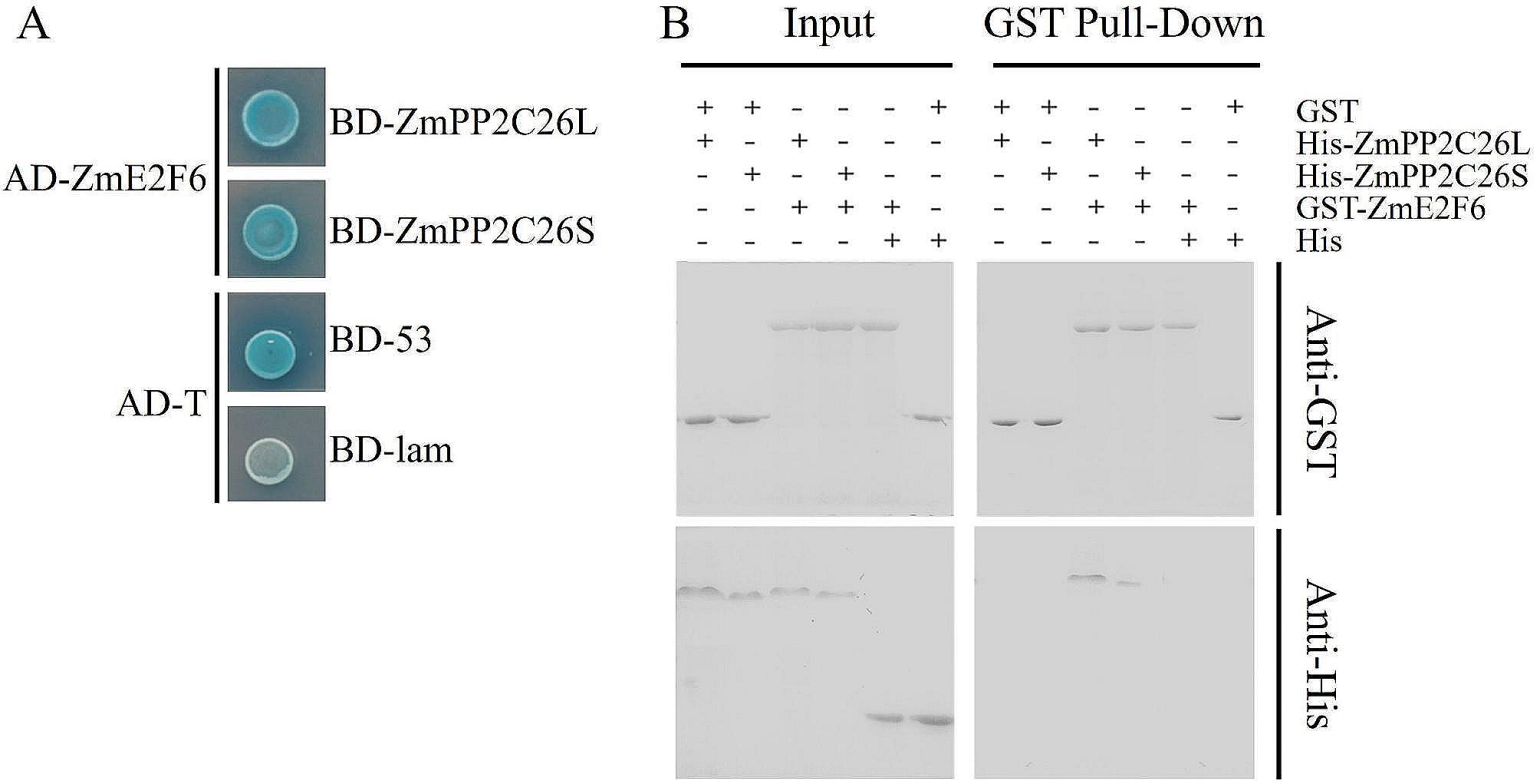
ZmE2F6 interacts with ZmPP2C26. (**A**) Yeast two-hybrid (Y2H). (**B**) GST pull-down. ZmPP2C26L and ZmPP2C26S represent two splicing variants of ZmPP2C26. The combination of AD-T with BD-53 and AD-T with BD-lam were used as negative and positive controls, respectively

In our previous study, ZmE2F6 was identified as a potential target of two ZmPP2C26 splicing variants (ZmPP2C26L/S) via Y2H library screening. Hence, to verify whether ZmE2F6 interacts with ZmPP2C26, Y2H, and GST pull-down assays were performed. As shown in Fig. 5, the yeast cells transformed with AD-*ZmE2F6* and BD-*ZmPP2C26L/S* exhibited normal growth and turned blue on SD/-Leu/-Trp/-His/-Ade plates containing X-α-gal. Moreover, the GST-ZmE2F6 protein could be pulled down by His-ZmPP2C26 L/S in the GST pull-down assay. These findings confirmed the interaction between ZmE2F6 and ZmPP2C26L/S. Likewise, there were 7 potential phosphorylated sites in ZmE2F6 amino acid sequences (Table S6) were predicted using NetPhos-3.1 (https://services.healthtech.dtu.dk/services/NetPhos-3.1/).

### ZmE2F6 localized to the nucleus

**Fig. 6:**
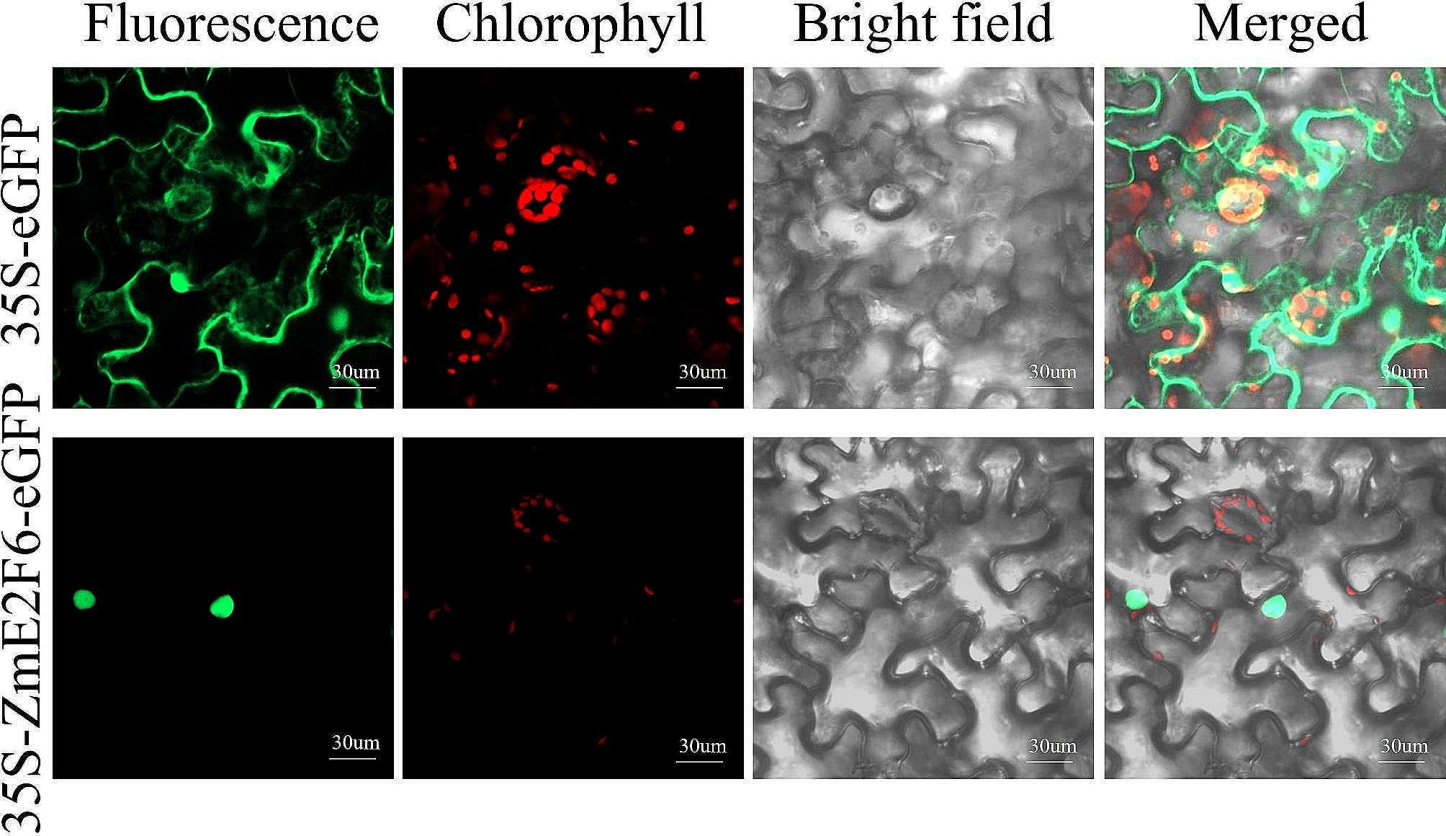
Subcellular localization of ZmE2Fs

To further validate the cellular localization of the ZmE2F6 protein, the ORF of *ZmE2F6* was cloned and inserted into the *35 S*-*eGFP* plasmid to fuse with *eGFP*. The results of transient expression in tobacco leaves revealed that fluorescent signals were observed in whole cells, including the nucleus and cytoplasm, transformed by the *35 S*-*eGFP* empty vector. However, in tobacco leaves transformed with *35 S*-*ZmE2F6*-*eGFP*, fluorescent signals were exclusively detected in the nucleus (Fig. 6). This observation is consistent with the bioinformatic prediction, confirming that the ZmE2F6 transcription factor is solely localized in the nucleus.

### The expression of *ZmE2F6* was induced by drought stress

**Fig. 7:**
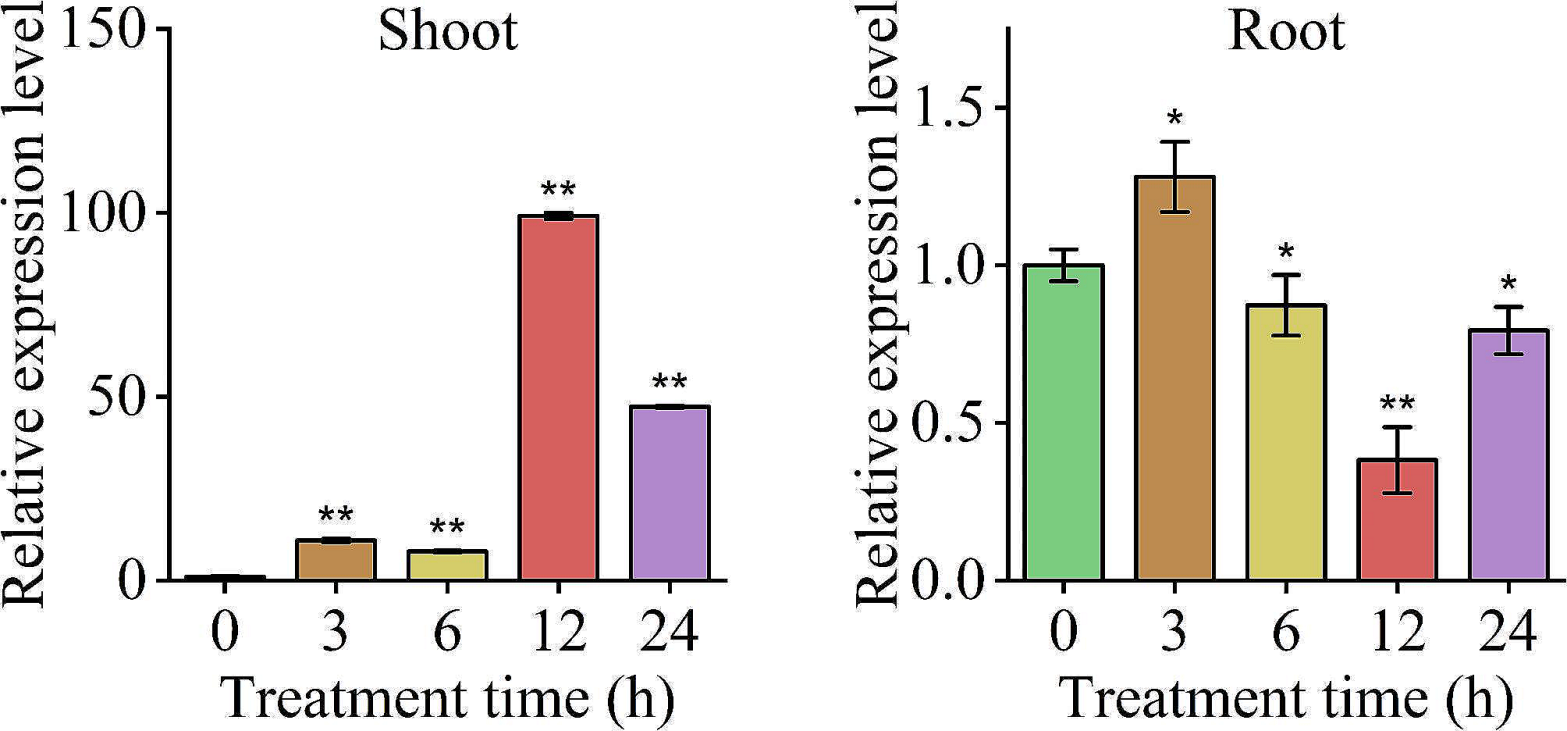
The relative expression level of the *ZmE2F* gene under drought stress

The qRT-PCR results demonstrated that the expression of the *ZmE2F6* gene was responsive to drought stress (Fig. 7). After drought treatment, the expression of *ZmE2F6* in maize shoots was significantly upregulated and reached approximately 11-, 8-, 100-, and 47-fold that of the control at 3, 6, 12, and 24 h of treatment. In roots, the expression of *ZmE2F6* was significantly upregulated after 3 h of drought treatment and then downregulated at 6, 12, and 24 h of treatment. These results suggest that *ZmE2F6* responds to drought stress.

### Expression of *ZmE2F6* enhanced drought tolerance in *Arabidopsis thaliana*

**Fig. 8:**
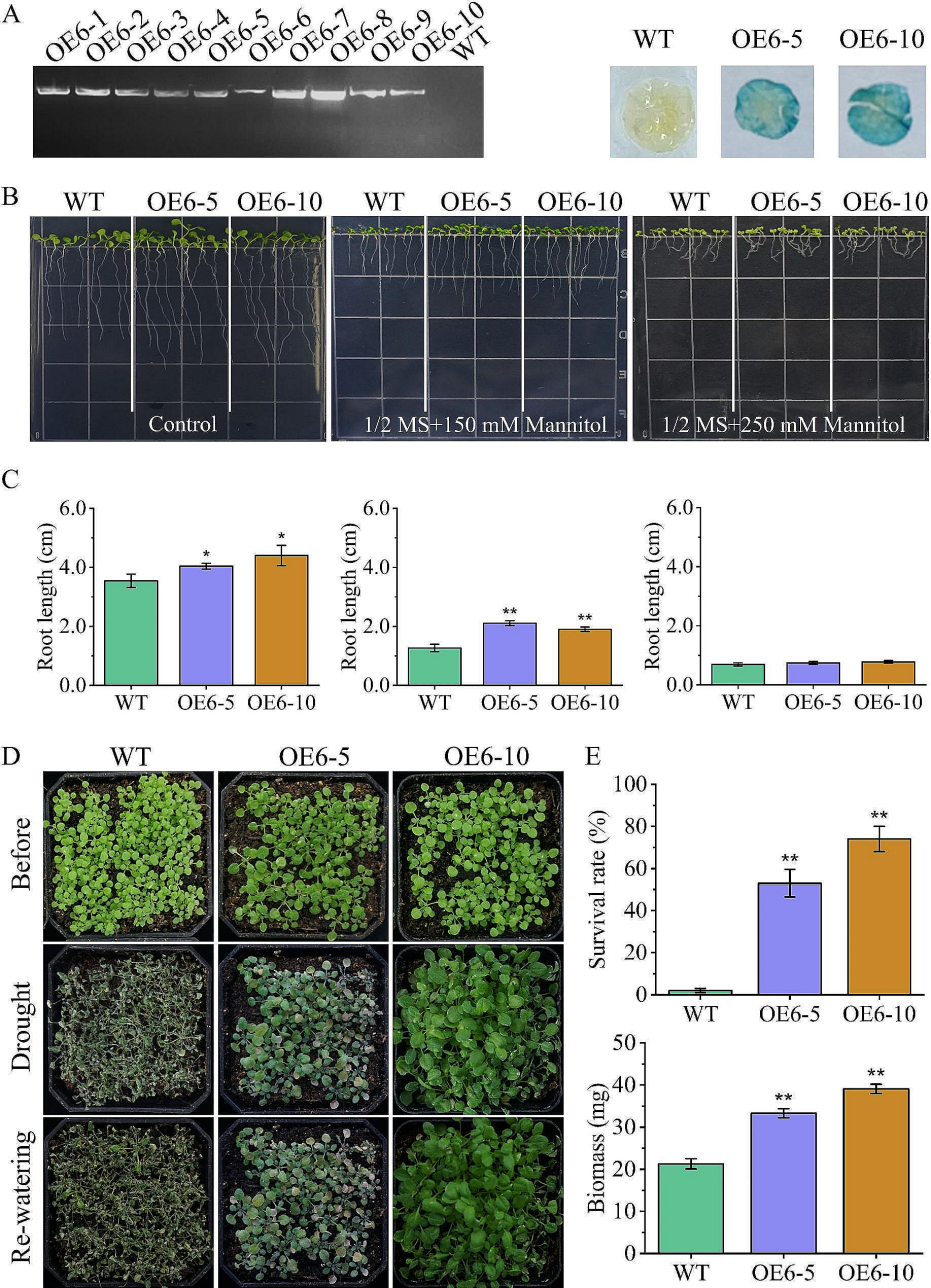
The phenotype of transgenic *Arabidopsis* under drought stress. (**A**) PCR detection and GUS staining of transgenic lines. (**B**) The phenotype of transgenic lines on 1/2 MS plates with mannitol. (**C**) Root length. (**D**) The phenotype of transgenic lines in soil. (**E**) Survival rate and biomass of each line. OE6-1 to OE6-10 represent transgenic lines overexpressing *ZmE2F6*. WT, wild type

To assess the function of ZmE2F6 in regulating drought tolerance, we generated transgenic *Arabidopsis* lines overexpressing *ZmE2F6*. In the T_1_ generation, ten positive transgenic lines were screened by kanamycin on 1/2 MS plates, identified by PCR, and harvested to produce the next generation. Finally, in the T_3_ generation, two homozygous lines (OE6-5 and OE6-10) were selected, identified by GUS staining, and used for phenotyping (Fig. 8A). It was found that the root length of the OE6-5 and OE6-10 lines was significantly longer than that of the WT under 1/2 MS plates or supplemented with 150 mM mannitol (Fig. 8B, C). Furthermore, natural drought treatment in soil was performed to monitor the drought tolerance of the OE6-5 and OE6-10 lines. The results showed that there was no difference between the transgenic lines and WT before treatment. Subsequently, after two weeks of drought treatment, WT plants were seriously wilting, but OE6-5 and OE6-10 showed slightly inhibited phenotypes. After 3 days of rewatering, the OE-65 and OE6-10 lines exhibited significantly higher survival rates and biomass, indicating that overexpression of *ZmE2F6* contributes to enhancing drought tolerance in transgenic *Arabidopsis* and that *ZmE2F6* positively regulates drought tolerance.

**Fig. 9:**
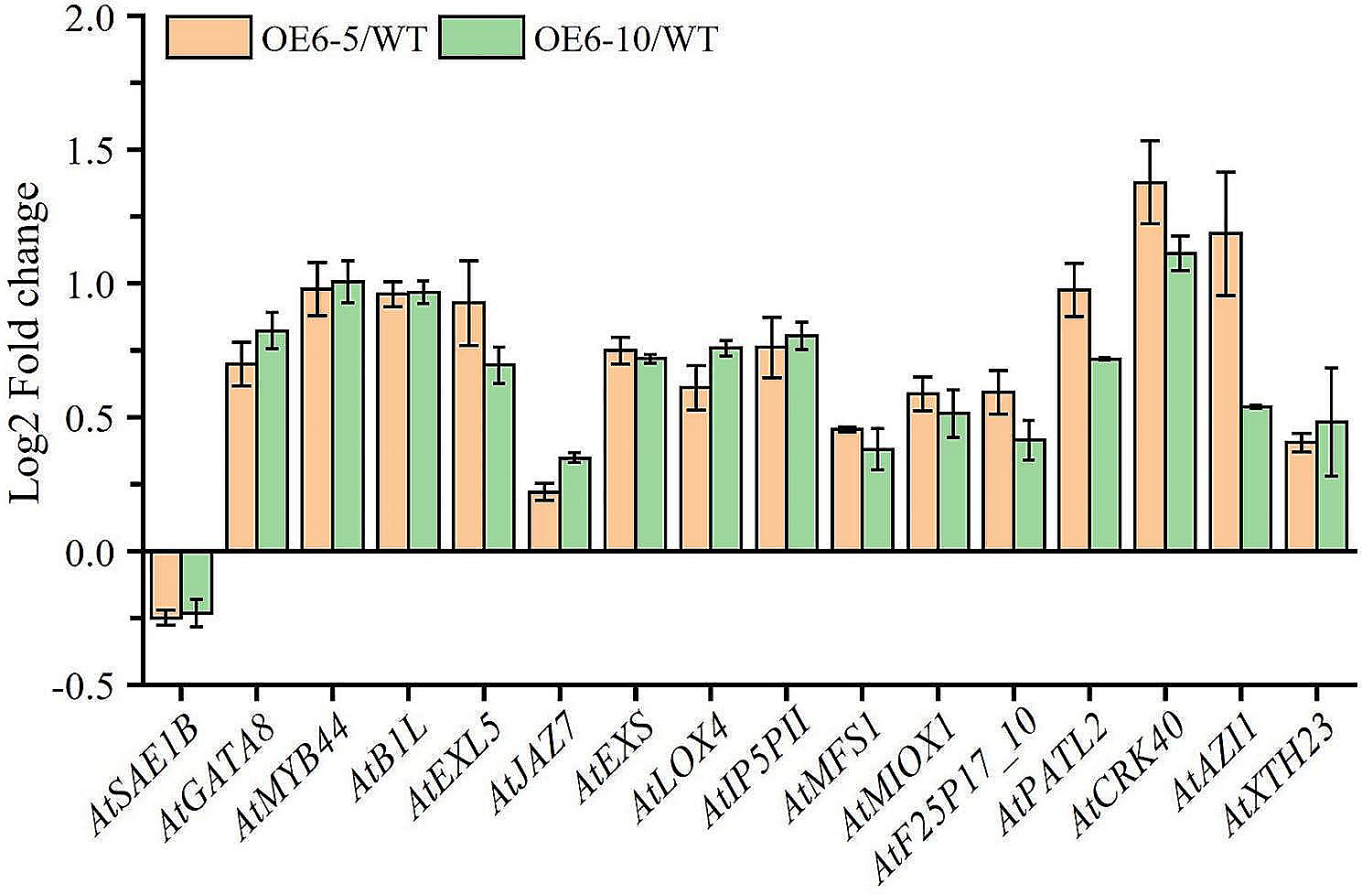
The expression level (Log2 Fold change) of differentially expressed genes (DEGs) in each line

To investigate the impact of ZmE2F6 overexpression on endogenous genes in *Arabidopsis thaliana*, RNA-Seq was conducted on four transgenic lines, OE6-5, OE6-10, and WT. The results showed that there were 657 DEGs in transgenic lines compared to WT. Totally, 19 DEGs were identified in two transgenic lines. Among them, 15 DEGs were upregulated in transgenic lines, including *AtMYB44* (AT5G67300), *AtB1L* (AT1G18740), *AtJAZ7* (AT2G34600), *AtEXS* (AT1G35350), *AtIP5PII* (AT4G18010), *AtPATL2* (AT1G22530), *AtAZI1* (AT4G12470), *AtXTH23* (AT4G25810), and *AtEXL5* (AT2G17230) (Fig. 9). GO analysis showed that these DEGs were associated with stress responses (Figure S1).

## Discussion

In eukaryotes, the ZmE2F family is characterized as a TF that plays crucial roles in cell division, DNA repair, and differentiation [11, 13–16]. However, the E2F family is only genome-wide identified in a few plants, including *Arabidopsis*, rice, wheat, Moso bamboo, *Medicago truncatula*, and *Phaseolus vulgaris* [9, 24–26]. In the present study, 21 *ZmE2F* members were identified in the maize genome (Table 1) and classified into E2F, DP, and DEL subclades (Fig. 1), which was higher than the number of E2F members identified in *Arabidopsis* (8), rice (9), *Medicago truncatula* (5), and *Phaseolus vulgaris* (7) but close to the number in wheat (27) and *Moso bamboo* (23), owing to their comparable genome size and gene duplication being a major driving force of gene families [25, 49]. Likewise, eight paralogous pairs of *ZmE2Fs* and twenty-three orthologous pairs of *E2Fs* among maize, *Arabidopsis*, and rice were found (Fig. 4). All the Ka/Ks ratios of the ZmE2Fs paralogous pairs were < 1 (Table S4), indicating their duplication evolved under purifying selection [50]. A similar phenomenon was consistently observed in the *PheE2F/DP* gene family [25]. A previous study showed that there are 12 *ZmE2F* genes in maize [51], which is much lower than the number identified in this study. This may be due to their preliminary BLAST search using maize genome release 5b.60 and not a comprehensive analysis [51].

All ZmE2F proteins possess at least one DBD characterized by motif 1 containing the RRIYD sequence and dimerization residue DNVLE sequence (Fig. 2), which contributes to the binding of E2F and DNA as a homodimer or as a heterodimer with its dimerization partner DP. In addition, some ZmE2F proteins also have DD and CC-MB domains, which promotes their formation of heterodimers to regulate downstream genes [52]. As a result, ten ZmE2Fs are predicted to interact with each other and generate 23 PPI combinations (Fig. 3), owing to the presence of the DNVLE residues, DD, or CC-MB domain in these proteins.

To date, the function of E2F in regulating plant stress tolerance remains unknown, although few reports have shown that the expression of some *E2F* genes is responsive to stress [9, 24–26]. In our study, abundant stress-responsive acting elements were found in *ZmE2F* promoter regions, such as ABREs and MBSs, suggesting the response of *ZmE2Fs* to stress and the potential roles of *ZmE2Fs*. In our previous study, the *ZmPP2C26* gene, a B clade of maize *PP2C* members, was found to be responsive to drought stress and negatively regulated drought tolerance in *Arabidopsis*, rice, and maize [36] and targeted on maize *ZmE2F6* (Zm00001d048412), indicating its function in the drought response. Hence, the *ZmE2F6* gene was cloned and functionally validated. It is found that the ZmE2F6 interacts with two splicing variants of ZmPP2C26 but localized in the nucleus (Figs. 5 and 6), suggesting that ZmPP2C26 physically targets ZmE2F6. In *Arabidopsis*, it has been previously reported that B clade PP2C (AP2C1) dephosphorylates the autophosphorylated form of CBL-interacting protein kinase 9 (CIPK9) to regulate root growth, seedling development, and stress tolerance (low-K^+^) [53]. Previous studies showed that BES1/BZR1 TFs are phosphorylated and degraded but moved to the nucleus after dephosphorylation in the cytoplasm [54, 55]. It is proposed that ZmPP2C26 might dephosphorylate ZmE2F6 in the cytoplasm, which needs to be further revealed in our next study.

The qRT-PCR results showed that the *ZmE2F6* gene was induced by drought stress (Fig. 7), which could be explained by the presence of three MBS and two ABREs in the *ZmE2F6* promoter (Table S5). It’s confirmed that MYB transcription factors bind to the MYB binding sites (MBS) of nuclear gene promoters to adjust their transcription and regulate drought tolerance in plants [56, 57]. Likewise, ABRE (ABA-responsive element), the major *cis*-element for ABA-responsive gene expression, is targeted by ABRE-binding protein (AREB) or ABRE-binding factor (ABF) TFs to regulate the drought response via the ABA signaling pathway [58]. These findings further imply the regulation of *ZmE2F6* in drought tolerance.

After overexpressing *ZmE2F6* in *Arabidopsis*, the transgenic lines showed longer root lengths than the WT (Fig. 8B, C), which is consistent with the fact that E2Fs regulate root growth in *Arabidopsis* [19, 22, 59]. The elevated root growth of *ZmE2F6*-overexpressing lines may confer osmotic tolerance because roots can respond to moisture and coordinate responses to drought [1]. The ectopic expression of *ZmE2F6* enhances drought tolerance in transgenic *Arabidopsis* (Fig. 8). We found that some stress-related genes were significantly upregulated in the transgenic lines, such as *AtMYB44*, *AtB1L*, *AtJAZ7*, *AtEXS*, *AtIP5PII*, *AtPATL2*, *AtAZI1*, *AtXTH23*, and *AtEXL5* (Fig. 9), which are well known to regulate abiotic stresses [60–68]. The ZmE2F6 protein acts as a TF and localizes in the nucleus to regulate the expression of these genes (Fig. 6). However, the molecular mechanism regulated by ZmE2F6 in maize remains unknown.

## Conclusion

Overall, in this study, a comprehensive analysis of the *ZmE2F* gene family was performed. In total, 21 ZmE2F TFs were identified in the maize genome and divided into three subclades. All ZmE2F proteins possessed at least one DBD characterized by the RRIYD (DNA binding motif) and DNVLE (dimerization residues) sequences. The *ZmE2F* genes showed diversity in gene structure, expanded by gene duplication, and contained abundant stress-responsive elements in their promoter regions. Then, the *ZmE2F6* gene was cloned and functionally verified in the drought response. The ZmE2F6 protein interacted with ZmPP2C26, localized in the nucleus, and responded to drought treatment. The overexpression of *ZmE2F6* enhanced drought tolerance in transgenic *Arabidopsis* by upregulating stress-related gene transcription. This study sheds light on the role of ZmE2F in the drought response and provides novel insights into a greater understanding of the E2F family in crops.

### Electronic supplementary material

Below is the link to the electronic supplementary material.

Supplementary Material 1

Supplementary Material 2

## Data Availability

The RNA-seq datasets generated during the current study are available in the NCBI repository under BioProject accession number PRJNA1028664.
