# Supplementary figures and images for: Comprehensive identification of maize ZmE2F transcription factors and the positive role of ZmE2F6 in response to drought stress
Source: BMC Genomics. 2024 May 13;25:465. doi: 10.1186/s12864-024-10369-0 (PMC11092242; doi:10.1186/s12864-024-10369-0)

Figure S1. GO results of DEGs.


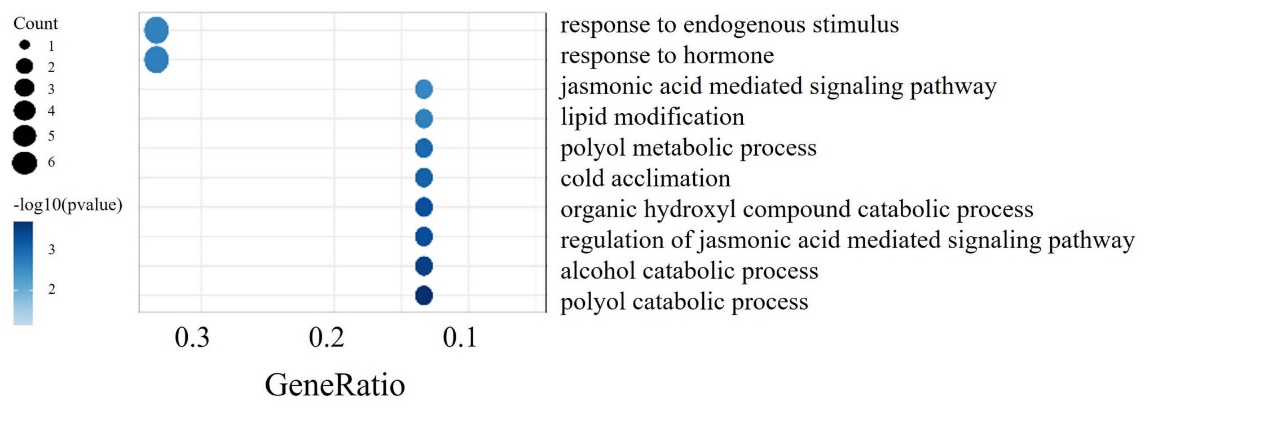


Figure 5B. Original figures


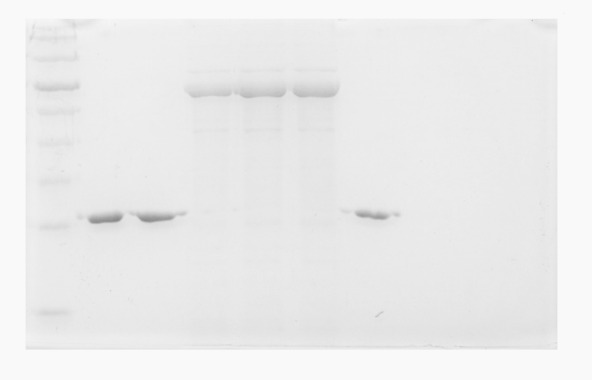

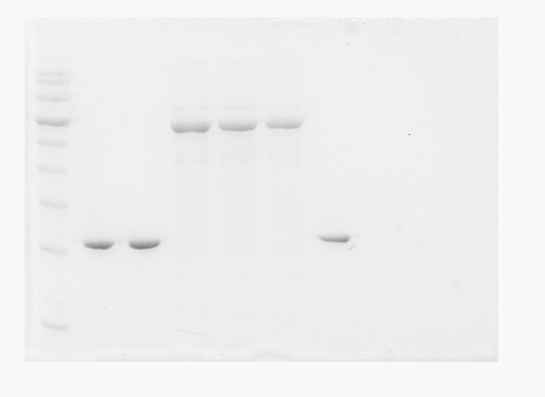


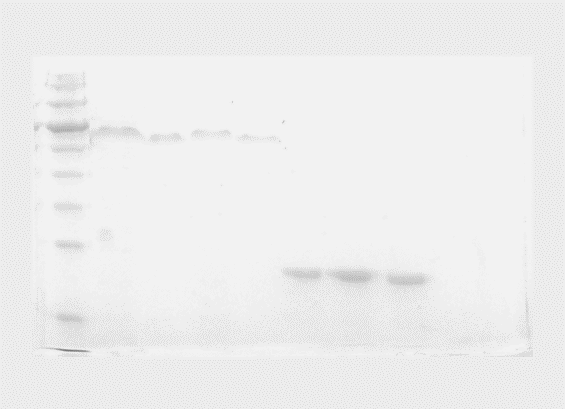

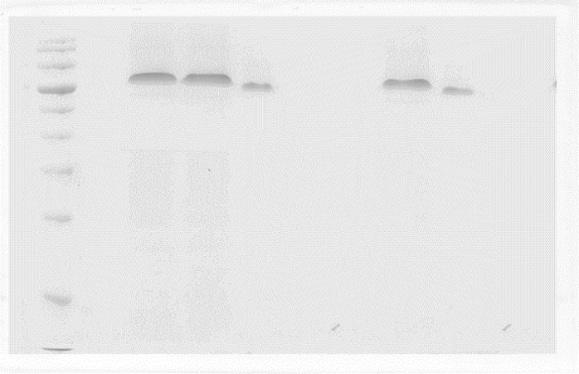


Figure 8A Original figure


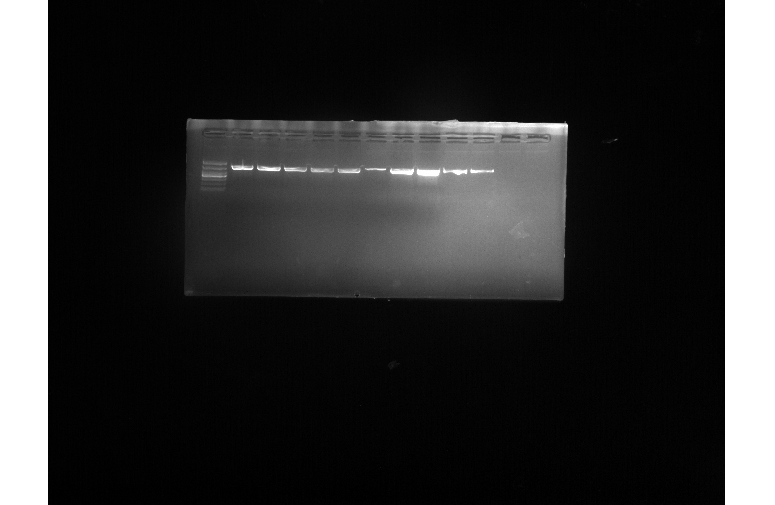

Supplement: Supplementary file 1 — Supplementary Material 1 [file 12864_2024_10369_MOESM1_ESM.docx]
