# Supplementary material for: Comprehensive identification of maize ZmE2F transcription factors and the positive role of ZmE2F6 in response to drought stress
Source: BMC Genomics. 2024 May 13;25:465. doi: 10.1186/s12864-024-10369-0 (PMC11092242; doi:10.1186/s12864-024-10369-0)
Supplement: Supplementary file 2 — Supplementary Material 2 [file 12864_2024_10369_MOESM2_ESM.docx]

| **Table S1** The primers used in the study | | |
| --- | --- | --- |
| **Primers** | **Sequences** | **Note** |
| ZmE2F6F | TTCCTCCGCTTGAATAATCTC | Gene cloning |
| ZmE2F6R | CATCTCCATACGCTGTGCTTG |  |
| ZmE2F6-QF | GGCTGGCTACACAGACTCTT | qRT-PCR |
| ZmE2F6-QR | GCAGTCCCCGGTTTCCTTTA |  |
| 2300-ZmE2F6-F | ggtacccggggatcctctagaATGGTCTCCGGCGCGGCG | plant transient expression vectors |
| 2300-ZmE2F6-R | cttgctcaccatggtactagtGTGCTGCTCGTGCTTCACACG |  |
| AD-E2F6F | tcagaggaggacctgcatatgATGGTCTCCGGCGCGGCG | Y2H |
| AD-E2F6R | atgcggccgctgcaggtcgacGTGCTGCTCGTGCTTCACACG |  |
| 32a-PP2C26LF | gctgatatcggatccgaattcATGTCTTGCACGGTGGCCA | The primers of prokaryotic expression vector. |
| 32a-PP2C26LR | ctcgagtgcggccgcaagcttTTACGAGCTTGAAAATTTCTGCA |  |
| 32a-PP2C26SF | gctgatatcggatccgaattcATGGACGGCGGGGGGCAG |  |
| 32a-PP2C26SR | ctcgagtgcggccgcaagcttCTACTGGTAATCTGGGTTGAATGC |  |
| 6p-E2F6F | cccctgggatccccggaattcATGGTCTCCGGCGCGGCG |  |
| 6p-E2F6R | gtcacgatgcggccgctcgagCTAGTGCTGCTCGTGCTTCACA |  |
| 201-E2F6F | ttctacaggacgtaacatatgATGGTCTCCGGCGCGGCG | Construction primers of plant overexpression vector. |
| 201-E2F6R | tcttcactgttgatacatatg GTGCTGCTCGTGCTTCACACG |  |

| **Table S2** The Pfam results of ZmE2F members | | |
| --- | --- | --- |
| **Gene name** | **Gene ID** | **Pfam ID** |
| *ZmE2F1* | Zm00001d032741 | PF02319 |
| *ZmE2F2* | Zm00001d007384 | PF02319 |
| *ZmE2F3* | Zm00001d033566 | PF02319 |
| *ZmE2F4* | Zm00001d016737 | PF02319, PF16421 |
| *ZmE2F5* | Zm00001d004512 | PF02319, PF16421 |
| *ZmE2F6* | Zm00001d048412 | PF02319, PF08781 |
| *ZmE2F7* | Zm00001d017986 | PF02319 |
| *ZmE2F8* | Zm00001d016907 | PF02319 |
| *ZmE2F9* | Zm00001d011597 | PF02319, PF08781 |
| *ZmE2F10* | Zm00001d045365 | PF02319 |
| *ZmE2F11* | Zm00001d023465 | PF02319, PF16421 |
| *ZmE2F12* | Zm00001d037274 | PF02319 |
| *ZmE2F13* | Zm00001d052288 | PF02319 |
| *ZmE2F14* | Zm00001d038664 | PF02319 |
| *ZmE2F15* | Zm00001d026355 | PF02319 |
| *ZmE2F16* | Zm00001d050664 | PF02319, PF16421 |
| *ZmE2F17* | Zm00001d003755 | PF02319, PF16421 |
| *ZmE2F18* | Zm00001d014132 | PF02319 |
| *ZmE2F19* | Zm00001d027709 | PF02319, PF08781 |
| *ZmE2F20* | Zm00001d045639 | PF02319 |

Note: PF02319, E2F_TDP domain. PF16421, E2F_CC-MB domain. PF08781, DP domain.

| **Table S3** Orthologous pairs of E2Fs between maize and *Arabidopsis*/rice | |
| --- | --- |
| **ZmE2F ID** | **AtE2F/OsE2F ID** |
| Zm00001d003755 | AT2G36010 |
| Zm00001d023465 | Os12t0158800 |
| Zm00001d003755 | Os12t0158800 |
| Zm00001d048412 | Os10t0440100 |
| Zm00001d014132 | Os10t0440100 |
| Zm00001d027709 | Os10t0440100 |
| Zm00001d017986 | Os06t0245900 |
| Zm00001d037274 | Os06t0245900 |
| Zm00001d016737 | Os04t0416100 |
| Zm00001d023465 | Os04t0416100 |
| Zm00001d050664 | Os04t0416100 |
| Zm00001d003755 | Os04t0416100 |
| Zm00001d004512 | Os04t0112200 |
| Zm00001d048412 | Os03t0152100 |
| Zm00001d014132 | Os03t0152100 |
| Zm00001d027709 | Os03t0152100 |
| Zm00001d017986 | Os02t0739700 |
| Zm00001d037274 | Os02t0739700 |
| Zm00001d052288 | Os02t0739700 |
| Zm00001d016737 | Os02t0537500 |
| Zm00001d050664 | Os02t0537500 |
| Zm00001d003755 | Os02t0537500 |
| Zm00001d011597 | Os01t0678700 |

| **Table S4** The Ka/Ks ratios and estimated divergence times for duplicate pairs of ZmE2Fs. | | | | |
| --- | --- | --- | --- | --- |
| **Duplication gene pair** | **Ka** | **Ks** | **Ka/Ks** | **Divergence time (Mya)** |
| *ZmE2F4* and *ZmE2F17* | 0.2945 | 1.1569 | 0.2545 | 88.99 |
| *ZmE2F6* and *ZmE2F18* | 0.2883 | 1.0585 | 0.2724 | 81.42 |
| *ZmE2F6* and *ZmE2F19* | 0.0568 | 0.2427 | 0.2339 | 18.67 |
| *ZmE2F7* and *ZmE2F12* | 0.2593 | 1.0807 | 0.2400 | 83.13 |
| *ZmE2F7* and *ZmE2F13* | 0.0754 | 0.2150 | 0.3505 | 16.54 |
| *ZmE2F11* and *ZmE2F17* | 0.3538 | 1.8109 | 0.1954 | 139.30 |
| *ZmE2F12* and *ZmE2F13* | 0.2476 | 1.0527 | 0.2352 | 80.98 |
| *ZmE2F18* and *ZmE2F19* | 0.3004 | 0.9266 | 0.3242 | 71.27 |

Note: Ka, non-synonymous. Ks, synonymous. Mya, million years ago.

| **Table S5** The *cis*-acting elements in *ZmE2F* promoters | | | |
| --- | --- | --- | --- |
| **Gene** | **Position (bp)** | **Element** | **Annotation** |
| *ZmE2F1* | 379 | ARE | cis-acting regulatory element essential for the anaerobic induction |
|  | 1520 | TC-rich repeats | cis-acting element involved in defense and stress responsiveness |
|  | 138 | MBS | MYB binding site involved in drought-inducibility |
|  | 849 | MBS | MYB binding site involved in drought-inducibility |
|  | 976 | MBS | MYB binding site involved in drought-inducibility |
|  | 509 | TGA-element | auxin-responsive element |
|  | 1591 | MRE | MYB binding site involved in light responsiveness |
|  | 460 | ABRE | cis-acting element involved in the abscisic acid responsiveness |
|  | 700 | ABRE | cis-acting element involved in the abscisic acid responsiveness |
| *ZmE2F2* | 1254 | TCA-element | cis-acting element involved in salicylic acid responsiveness |
|  | 339 | MBS | MYB binding site involved in drought-inducibility |
|  | 958 | MBS | MYB binding site involved in drought-inducibility |
|  | 1142 | MBS | MYB binding site involved in drought-inducibility |
|  | 1771 | MBS | MYB binding site involved in drought-inducibility |
|  | 971 | ABRE | cis-acting element involved in the abscisic acid responsiveness |
|  | 745 | TC-rich repeats | cis-acting element involved in defense and stress responsiveness |
| *ZmE2F3* | 224 | ABRE | cis-acting element involved in the abscisic acid responsiveness |
|  | 226 | ABRE | cis-acting element involved in the abscisic acid responsiveness |
|  | 902 | ABRE | cis-acting element involved in the abscisic acid responsiveness |
|  | 1382 | TCA-element | cis-acting element involved in salicylic acid responsiveness |
|  | 410 | TGA-element | auxin-responsive element |
| *ZmE2F4* | 192 | ABRE | cis-acting element involved in the abscisic acid responsiveness |
|  | 695 | ABRE | cis-acting element involved in the abscisic acid responsiveness |
|  | 774 | ABRE | cis-acting element involved in the abscisic acid responsiveness |
|  | 853 | ABRE | cis-acting element involved in the abscisic acid responsiveness |
|  | 854 | ABRE | cis-acting element involved in the abscisic acid responsiveness |
|  | 1381 | ABRE | cis-acting element involved in the abscisic acid responsiveness |
|  | 822 | MSA-like | cis-acting element involved in cell cycle regulation |
|  | 21 | TGA-element | auxin-responsive element |
|  | 1341 | MBS | MYB binding site involved in drought-inducibility |
|  | 164 | ARE | cis-acting regulatory element essential for the anaerobic induction |
| *ZmE2F5* | 1242 | ARE | cis-acting regulatory element essential for the anaerobic induction |
|  | 1429 | ARE | cis-acting regulatory element essential for the anaerobic induction |
|  | 1584 | ARE | cis-acting regulatory element essential for the anaerobic induction |
|  | 607 | ABRE | cis-acting element involved in the abscisic acid responsiveness |
|  | 1783 | ABRE | cis-acting element involved in the abscisic acid responsiveness |
|  | 168 | MBS | MYB binding site involved in drought-inducibility |
|  | 988 | MBS | MYB binding site involved in drought-inducibility |
|  | 1123 | TGA-element | auxin-responsive element |
| *ZmE2F6* | 1537 | ABRE | cis-acting element involved in the abscisic acid responsiveness |
|  | 1694 | ABRE | cis-acting element involved in the abscisic acid responsiveness |
|  | 1293 | MRE | MYB binding site involved in light responsiveness |
|  | 1266 | TCA-element | cis-acting element involved in salicylic acid responsiveness |
|  | 1415 | TCA-element | cis-acting element involved in salicylic acid responsiveness |
|  | 425 | MBS | MYB binding site involved in drought-inducibility |
|  | 670 | MBS | MYB binding site involved in drought-inducibility |
|  | 680 | MBS | MYB binding site involved in drought-inducibility |
| *ZmE2F7* | 270 | TGA-element | auxin-responsive element |
|  | 1280 | TGA-element | auxin-responsive element |
|  | 799 | MBS | MYB binding site involved in drought-inducibility |
|  | 976 | MBS | MYB binding site involved in drought-inducibility |
|  | 823 | ABRE | cis-acting element involved in the abscisic acid responsiveness |
|  | 1356 | ABRE | cis-acting element involved in the abscisic acid responsiveness |
|  | 1422 | ABRE | cis-acting element involved in the abscisic acid responsiveness |
|  | 806 | LTR | cis-acting element involved in low-temperature responsiveness |
| *ZmE2F8* | 409 | TGA-element | auxin-responsive element |
|  | 1693 | MBS | MYB binding site involved in drought-inducibility |
|  | 1382 | TCA-element | cis-acting element involved in salicylic acid responsiveness |
|  | 223 | ABRE | cis-acting element involved in the abscisic acid responsiveness |
|  | 225 | ABRE | cis-acting element involved in the abscisic acid responsiveness |
|  | 901 | ABRE | cis-acting element involved in the abscisic acid responsiveness |
| *ZmE2F9* | 648 | ARE | cis-acting regulatory element essential for the anaerobic induction |
|  | 1500 | ARE | cis-acting regulatory element essential for the anaerobic induction |
|  | 1655 | ARE | cis-acting regulatory element essential for the anaerobic induction |
|  | 682 | ABRE | cis-acting element involved in the abscisic acid responsiveness |
|  | 1911 | ABRE | cis-acting element involved in the abscisic acid responsiveness |
|  | 1858 | MSA-like | cis-acting element involved in cell cycle regulation |
| *ZmE2F10* | 1491 | ARE | cis-acting regulatory element essential for the anaerobic induction |
|  | 1077 | ABRE | cis-acting element involved in the abscisic acid responsiveness |
|  | 1267 | ABRE | cis-acting element involved in the abscisic acid responsiveness |
|  | 1726 | ABRE | cis-acting element involved in the abscisic acid responsiveness |
|  | 1377 | LTR | cis-acting element involved in low-temperature responsiveness |
|  | 278 | MBS | MYB binding site involved in drought-inducibility |
| *ZmE2F11* | 1408 | LTR | cis-acting element involved in low-temperature responsiveness |
|  | 1633 | LTR | cis-acting element involved in low-temperature responsiveness |
|  | 362 | ABRE | cis-acting element involved in the abscisic acid responsiveness |
|  | 1395 | ABRE | cis-acting element involved in the abscisic acid responsiveness |
|  | 1396 | ABRE | cis-acting element involved in the abscisic acid responsiveness |
|  | 997 | TCA-element | cis-acting element involved in salicylic acid responsiveness |
|  | 583 | TGA-element | auxin-responsive element |
|  | 404 | MBS | MYB binding site involved in drought-inducibility |
|  | 1670 | TC-rich repeats | cis-acting element involved in defense and stress responsiveness |
|  | 1854 | TC-rich repeats | cis-acting element involved in defense and stress responsiveness |
|  | 770 | ARE | cis-acting regulatory element essential for the anaerobic induction |
|  | 951 | ARE | cis-acting regulatory element essential for the anaerobic induction |
|  | 1341 | ARE | cis-acting regulatory element essential for the anaerobic induction |
| *ZmE2F12* | 1162 | ARE | cis-acting regulatory element essential for the anaerobic induction |
|  | 1429 | ARE | cis-acting regulatory element essential for the anaerobic induction |
|  | 1170 | TGA-element | auxin-responsive element |
|  | 1251 | TGA-element | auxin-responsive element |
|  | 327 | MBS | MYB binding site involved in drought-inducibility |
|  | 40 | ABRE | cis-acting element involved in the abscisic acid responsiveness |
|  | 845 | MRE | MYB binding site involved in light responsiveness |
| *ZmE2F13* | 463 | ARE | cis-acting regulatory element essential for the anaerobic induction |
|  | 1093 | MSA-like | cis-acting element involved in cell cycle regulation |
|  | 907 | ABRE | cis-acting element involved in the abscisic acid responsiveness |
|  | 1388 | ABRE | cis-acting element involved in the abscisic acid responsiveness |
|  | 1389 | ABRE | cis-acting element involved in the abscisic acid responsiveness |
|  | 1442 | ABRE | cis-acting element involved in the abscisic acid responsiveness |
|  | 1443 | ABRE | cis-acting element involved in the abscisic acid responsiveness |
|  | 1966 | ABRE | cis-acting element involved in the abscisic acid responsiveness |
|  | 1981 | ABRE | cis-acting element involved in the abscisic acid responsiveness |
|  | 1982 | ABRE | cis-acting element involved in the abscisic acid responsiveness |
|  | 8 | MBS | MYB binding site involved in drought-inducibility |
|  | 1395 | MBS | MYB binding site involved in drought-inducibility |
|  | 846 | TCA-element | cis-acting element involved in salicylic acid responsiveness |
| *ZmE2F14* | 748 | TC-rich repeats | cis-acting element involved in defense and stress responsiveness |
|  | 1258 | TCA-element | cis-acting element involved in salicylic acid responsiveness |
|  | 342 | MBS | MYB binding site involved in drought-inducibility |
|  | 961 | MBS | MYB binding site involved in drought-inducibility |
|  | 1772 | MBS | MYB binding site involved in drought-inducibility |
|  | 974 | ABRE | cis-acting element involved in the abscisic acid responsiveness |
| *ZmE2F15* | 166 | TC-rich repeats | cis-acting element involved in defense and stress responsiveness |
|  | 154 | ABRE | cis-acting element involved in the abscisic acid responsiveness |
|  | 353 | ABRE | cis-acting element involved in the abscisic acid responsiveness |
|  | 1278 | ABRE | cis-acting element involved in the abscisic acid responsiveness |
|  | 98 | LTR | cis-acting element involved in low-temperature responsiveness |
|  | 307 | MBS | MYB binding site involved in drought-inducibility |
|  | 378 | MBS | MYB binding site involved in drought-inducibility |
|  | 1175 | MBS | MYB binding site involved in drought-inducibility |
| *ZmE2F16* | 29 | ABRE | cis-acting element involved in the abscisic acid responsiveness |
|  | 764 | ABRE | cis-acting element involved in the abscisic acid responsiveness |
|  | 817 | ABRE | cis-acting element involved in the abscisic acid responsiveness |
|  | 1817 | ABRE | cis-acting element involved in the abscisic acid responsiveness |
|  | 1438 | TGA-element | auxin-responsive element |
|  | 1056 | TCA-element | cis-acting element involved in salicylic acid responsiveness |
| *ZmE2F17* | 4 | ABRE | cis-acting element involved in the abscisic acid responsiveness |
|  | 5 | ABRE | cis-acting element involved in the abscisic acid responsiveness |
|  | 126 | ABRE | cis-acting element involved in the abscisic acid responsiveness |
|  | 1471 | ABRE | cis-acting element involved in the abscisic acid responsiveness |
|  | 666 | LTR | cis-acting element involved in low-temperature responsiveness |
|  | 103 | TGA-element | auxin-responsive element |
|  | 1069 | TC-rich repeats | cis-acting element involved in defense and stress responsiveness |
| *ZmE2F18* | 1146 | TCA-element | cis-acting element involved in salicylic acid responsiveness |
|  | 1071 | TGA-element | auxin-responsive element |
|  | 713 | ABRE | cis-acting element involved in the abscisic acid responsiveness |
|  | 714 | ABRE | cis-acting element involved in the abscisic acid responsiveness |
|  | 1668 | ABRE | cis-acting element involved in the abscisic acid responsiveness |
|  | 324 | ARE | cis-acting regulatory element essential for the anaerobic induction |
|  | 1840 | ARE | cis-acting regulatory element essential for the anaerobic induction |
| *ZmE2F19* | 1114 | LTR | cis-acting element involved in low-temperature responsiveness |
|  | 1885 | LTR | cis-acting element involved in low-temperature responsiveness |
|  | 394 | ABRE | cis-acting element involved in the abscisic acid responsiveness |
|  | 396 | ABRE | cis-acting element involved in the abscisic acid responsiveness |
|  | 561 | ABRE | cis-acting element involved in the abscisic acid responsiveness |
|  | 631 | ABRE | cis-acting element involved in the abscisic acid responsiveness |
|  | 904 | ABRE | cis-acting element involved in the abscisic acid responsiveness |
|  | 1006 | ABRE | cis-acting element involved in the abscisic acid responsiveness |
|  | 1511 | ABRE | cis-acting element involved in the abscisic acid responsiveness |
|  | 1557 | ABRE | cis-acting element involved in the abscisic acid responsiveness |
|  | 212 | ARE | cis-acting regulatory element essential for the anaerobic induction |
|  | 411 | ARE | cis-acting regulatory element essential for the anaerobic induction |
| *ZmE2F20* | 1281 | ARE | cis-acting regulatory element essential for the anaerobic induction |
|  | 1673 | TGA-element | auxin-responsive element |
|  | 617 | MBS | MYB binding site involved in drought-inducibility |
|  | 1487 | LTR | cis-acting element involved in low-temperature responsiveness |
|  | 660 | ABRE | cis-acting element involved in the abscisic acid responsiveness |
|  | 697 | ABRE | cis-acting element involved in the abscisic acid responsiveness |
|  | 772 | ABRE | cis-acting element involved in the abscisic acid responsiveness |
|  | 1527 | ABRE | cis-acting element involved in the abscisic acid responsiveness |
| *ZmE2F21* | 1197 | ARE | cis-acting regulatory element essential for the anaerobic induction |
|  | 1374 | ARE | cis-acting regulatory element essential for the anaerobic induction |
|  | 1571 | MBS | MYB binding site involved in drought-inducibility |
|  | 1733 | ABRE | cis-acting element involved in the abscisic acid responsiveness |

| **Table S6** Prediction results of phosphorylation sites of ZmE2F6 by kinase. | | | | |
| --- | --- | --- | --- | --- |
| **Protein** | **Position (aa)** | **Residue** | **Score** | **Kinase** |
| ZmE2F6 | 130 | S | 0.860 | PKC |
| ZmE2F6 | 388 | T | 0.860 | PKC |
| ZmE2F6 | 228 | S | 0.718 | PKC |
| ZmE2F6 | 17 | T | 0.705 | PKC |
| ZmE2F6 | 21 | S | 0.683 | cdk5 |
| ZmE2F6 | 112 | T | 0.651 | PKC |
| ZmE2F6 | 357 | T | 0.641 | DNAPK |

Note: aa, amino acid. S, serine. T, Threonine. PKC, Protein Kinase C. cdk5, cyclin-dependent kinase 5. DANPK, DNA-dependent protein kinase
